# Mycoendophytic-Derived Green Resveratrol-Conjugated Silver Nanoparticles Inhibit the Proliferation of Human Epidermoid Carcinoma A-431 Cells

**DOI:** 10.3390/ph19050656

**Published:** 2026-04-22

**Authors:** Amal A. Al Mousa, Mohamed E. Abouelela, Ahmed A. El-Shenawy, M. A. Abo-Kadoum, Adel Eltoukhy, Youssef Abo-Dahab, Rasha M. Allam, Nageh F. Abo-Dahab, Abdallah M. A. Hassane, Mohamed S. Refaey

**Affiliations:** 1 Department of Botany and Microbiology, College of Science, King Saud University, P.O. Box 145111, Riyadh 4545, Saudi Arabia; 2 Department of Pharmacognosy, Faculty of Pharmacy (Boys), Al-Azhar University, Cairo 11884, Egypt; m_abouelela@azhar.edu.eg; 3 Department of Pharmaceutics and Pharmaceutical Technology, Faculty of Pharmacy, Al-Azhar University, Assiut 71524, Egypt; ahmedsaleh@azhar.edu.eg; 4 Al-Azhar Centre of Nano Sciences and Applications, Al-Azhar University, Assiut 71524, Egypt; 5 Department of Pharmaceutics and Pharmaceutical Technology, Michael Sayegh Faculty of Pharmacy, Aqaba University of Technology, Aqaba 11191, Jordan; 6 Botany and Microbiology Department, Faculty of Science, Al-Azhar University, Assiut 71524, Egypt; mohamedabdellah@azhar.edu.eg (M.A.A.-K.); adelaly@azhar.edu.eg (A.E.); abodahabn@azhar.edu.eg (N.F.A.-D.); 7 Department of Bioengineering and Therapeutic Sciences, Schools of Pharmacy and Medicine, University of California, San Francisco 1700 Fourth St, San Francisco, CA 94158, USA; youssef.abo-dahab@ucsf.edu; 8 Pharmacology Department, Medical and Clinical Research Institute, National Research Centre, Dokki, Cairo 12622, Egypt; rasha_senior@yahoo.com; 9 Department of Pharmacognosy, Faculty of Pharmacy, University of Sadat City, Sadat City 32897, Egypt; mohamed.said@fop.usc.edu.eg; 10 Department of Pharmacognosy and Natural Products, Faculty of Pharmacy, Menoufia National University, km Cairo-Alexandria Agricultural Road, Shebin El-Kom 32511, Egypt

**Keywords:** *Alternaria alternata*, resveratrol, silver nanoparticles, A-431 cells, cytotoxicity

## Abstract

**Background/Objectives**: Endophytic fungi represent an alternative source for resveratrol (RES) production. The present study aims to utilize mycoendophytic-derived resveratrol as a reducing agent for the synthesis of silver nanoparticles (AgNPs), in addition to further assay the cytotoxic activity of a RES-conjugated nanocarrier system toward human epidermoid carcinoma A-431 cells. **Methods**: *Alternaria alternata* AUMC 16209 was isolated from the stem of grapevine *Vitis vinifera* L. cultivar prime. Strain identification was achieved through morphological and molecular characterization using ITS sequencing. *A. alternata* AUMC 16209 exhibited RES production capability upon cultivation on PDB medium for seven days with a total of 8.25 mg/L as determined by HPLC. The crude RES was purified using flash chromatography followed by structure elucidation through ^1^H and ^13^C NMR analyses. The purified RES was used for green synthesis of nanoparticles, acting as a reducing agent for silver ions. **Results**: Stable RES-AgNPs were fabricated at particle sizes ranging from 25 to 47 nm. RES-AgNPs observed a plasmon resonance absorption band at 415 nm with a negative zeta potential value of −38.5 mV. The crystalline structure of RES-AgNPs was addressed through X-ray diffraction analysis. FT-IR spectroscopy confirms the involvement of the functional –OH group and the aromatic C=C bond in the reduction and stabilization process. RES-AgNPs was more efficient to inhibit the cellular proliferation of human epidermoid carcinoma A-431 cells compared to RES alone. **Conclusions**: This report introduces for the first time an endophytic *A. alternata* as a sustainable source for RES production and emphasizes its potential for green synthesis of stable AgNPs with promising cytotoxic activity.

## 1. Introduction

Resveratrol is a polyphenolic stilbene compound ubiquitously found in grapes (*Vitis vinifera* L.) and other plants, acting as a phytoalexin, protecting them from both biotic and abiotic stress. Grapes attract a lot of attention owing to their highest concentration of RES compared to other plants. Due to its tremendous properties and applications, a high-RES quantity is urgently required to fulfill the world demand. RES was traditionally obtained from plant sources; however, this method requires precise environmental management and a long cultivation period with low yield [1]. Grape-associated fungi possess the potential to produce secondary metabolites like those of their host plants [2]. Several fungal endophytes isolated from grapes (*Vitis vinifera* L.) have been reported to produce resveratrol, such as *Aspergillus* sp., *Penicillium* sp., *Alternaria* sp., *Nigrospora* sp., *Fusarium* sp., and *Xylaria* sp. [2,3,4]. Overall, endophytic fungi represent a sustainable reservoir for RES production as an alternative to plant origin due to their easy and faster cultivation, high fermentation scalability, manipulation of biosynthetic pathways, and strains diversity [5].

RES has drawn attention due to its diverse applications in medicine, food processing, agriculture, pharmaceuticals, and cosmetics. Of particular interest, RES has therapeutic potential as cardiovascular protection, antioxidant, anti-inflammatory, and anti-tumor agents. All these applications are attributed to its ability to interfere with multiple signaling pathways and regulate metabolic events, thereby dictating disease processes [6,7]. RES exerts little side effects on adjacent normal tissues compared to routinely used chemotherapeutic agents [8]. Preclinical investigations highlighted the multifaceted potential of RES in cancer treatment. RES suppresses cancer cell proliferation and metastasis through activating apoptotic signaling pathways and modulating tumor-suppressive signaling NF-κB, PI3K/AKT, and p53 [9]. RES was reported to inhibit breast cancer cell prognosis via inducing apoptosis and enhancing chemotherapeutic efficacy [10]. Similarly, in prostate cancer, RES was found to restrain cell proliferation and activate apoptotic signals [11]. The activity of RES toward epidermoid carcinoma has been documented in several reports. RES exhibited antiproliferative capacity against human epidermoid carcinoma A-431 cells through inhibition of MEK1, (ERK) 1/2, and AP-1 signaling routs [12]. Zhang et al. reported that RES improved treatment output of photodynamic therapy for squamous cell carcinoma (SCC) and suppressed the proliferation of A-431 cells via activation of caspases cascade [13].

The therapeutic potential of RES is largely undermined due to low oral bioavailability, rapid metabolism, poor water solubility, instability, and high sensitivity [14]. Oral administration of RES in humans resulted in rapid metabolism with low bioavailability of unmetabolized form [15]. During the last two decades, several efforts have been implemented to ensure optimum RES delivery. Incorporating nanotechnology with RES can overcome these obstacles and enhance its therapeutic potential. RES has medicinal potential, but its fast metabolic breakdown and poor water solubility prevent it from being used in clinical settings. Although studies on nanocarriers frequently employ synthetic RES, endophytic fungi as sustainable bio-factories have gained attention recently due to developments in green nanotechnology and the circular bio-economy [16,17].

*Mentha* L., *Ginkgo biloba* L., and *Origanum* L. are among the medicinal plants that have been successfully used to produce AgNPs. AgNP biosynthesis techniques that make use of plant extracts have grown in importance. Even though the biosynthetic method using plant extracts has been well studied, its drawbacks—such as the lengthy development cycle of plants and the difficulties involved in large-scale reproduction—remain [18,19,20]. Researchers have focused a lot of emphasis on microbial-mediated synthesis of metal nanoparticles (MNPs) due to its ease of mass manufacturing and low cost of cultivation. It has been shown that a variety of bacteria, such as *Escherichia coli*, *Bacillus*, *Aspergillus*, and *Penicillium*, may synthesize AgNPs [21,22,23]. Compared to other microorganisms, endophytic fungi have greater promise in the field of MNPs production. According to a study by Sahana and Udayashankara [24], fungi typically exhibit higher efficacy than bacteria during the production of AgNPs because of their inherent metal accumulating characteristics and superior tolerance. As a valuable class of biological resources, endophytic fungus from medicinal plants can create additional bioactive compounds with pharmacological potential. Nevertheless, there is still a large research gap regarding the use of endophytic fungi from medicinal plants to synthesize AgNPs, and they have not yet been completely utilized [25].

Nanocarrier systems protect RES from enzymatic degradation and augment its bioavailability [26]. RES-conjugated nanoparticles (NPs) have been widely evaluated as promising strategies to improve their anti-tumor efficacy. RES-conjugated phytosome nanoparticles significantly reduced tumor volume in albino mice inoculated with Ehrlich ascites carcinoma (EAC) compared to free RES [27]. RES-loaded gold nanoparticles were more efficient for inhibition of pancreatic cancer cell line (AsPC-1) proliferation compared to individual application of RES and gold nanoparticles [28]. Recent reports revealed that RES-AgNPs significantly alleviate oxidative stress and inflammation in a rat model compared to the free RES form [29]. One special benefit of fungus-derived RES, such as that from *A. alternata* (Fries) Keissler, is the inclusion of biogenic co-metabolites and proteins that can serve as organic stabilizing and capping agents during the creation of nanoparticles. This work aimed to close the gap between nano-oncology and endophytic secondary metabolism. In particular, we created RES-conjugated silver nanoparticles and isolated a high-yield RES-producing strain of *A. alternata* (AUMC 16209) from *Vitis vinifera*. In contrast to conventional delivery techniques, we predicted that this biogenic conjugation would improve the anti-tumor efficacy against human epidermoid carcinoma (A-431), offering a first-of-its-kind report on the oncological potential of endophyte-sourced RES-nanohybrids.

## 2. Results and Discussion

### 2.1. Molecular Identification of A. alternata

**Figure 1:**
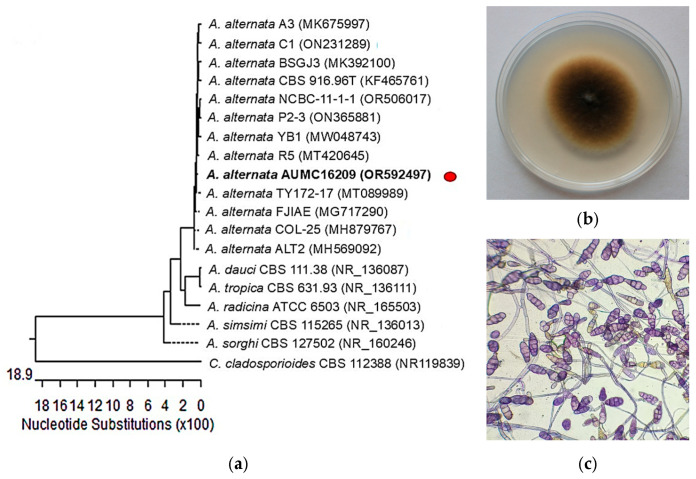
(**a**) The neighbor-joining phylogenetic tree based on the ITS gene sequences of the strain *A. alternata* AUMC 16209 with closely related strains accessed from the GenBank utilizing BLASTN; (**b**) morphological features of AUMC 16209 strain on potato dextrose agar (PDA); (**c**) AUMC 16209 examined by optical microscope.

Identification of the endophytic *A. alternata* (Fries) Keissler isolate (Figure 1) was performed morphologically and molecularly by the ITS region sequencing, and sequences were further subjected to a BLAST+ 2.17.0 search at the NCBI database. The isolate was confirmed as *A. alternata* AUMC 16209 (GenBank accession no. OR592497). Nucleotide comparisons among similar *Alternaria* strains’ ITS regions recovered from NCBI revealed 99.82% identity and 99% coverage with many strains of the same species including the type material *A. alternata* CBS 916.96^T^ with GenBank accession no. KF465761. A representative strain of *Cladosporium cladosporioides* is included as an outgroup species. Genus *Alternaria* is well known as a natural occurring grape endophyte with constitutive potential for the production of RES and its derivatives [30]. *Alternaria* sp. MG1 has been previously isolated from the cob of *Vitis vinifera* L. and exhibited stable RES production over multiple subcultures on glucose medium [31]. Transcriptomic analysis proved the presence of RES biosynthesis pathway in *Alternaria* sp. MG1, similar to that found in plants [32]. The ITS regions represent an important efficient marker for confirming fungal strains identification at species level [33]. Abdel-Hadi et al. [34] characterized RES-producing *A. alternata* SK from Brinjal based on ITS sequencing.

### 2.2. Detection, Purification and Identification of RES

**Figure 2:**
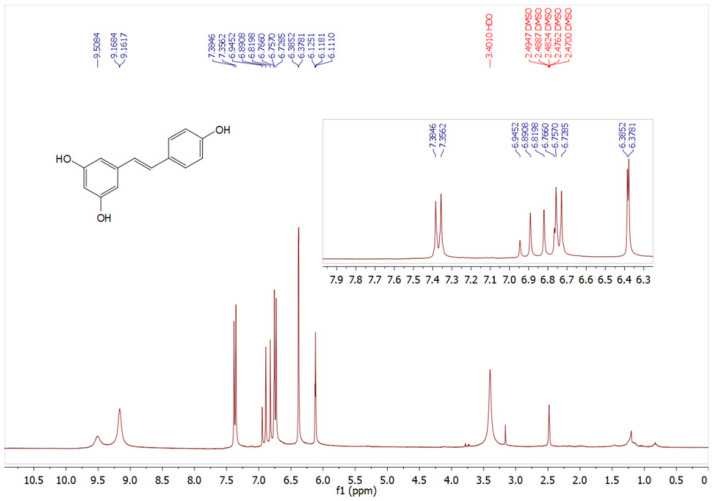
^1^H-NMR of resveratrol (DMSO-*d*_6_, 300 MHz).

**Figure 3:**
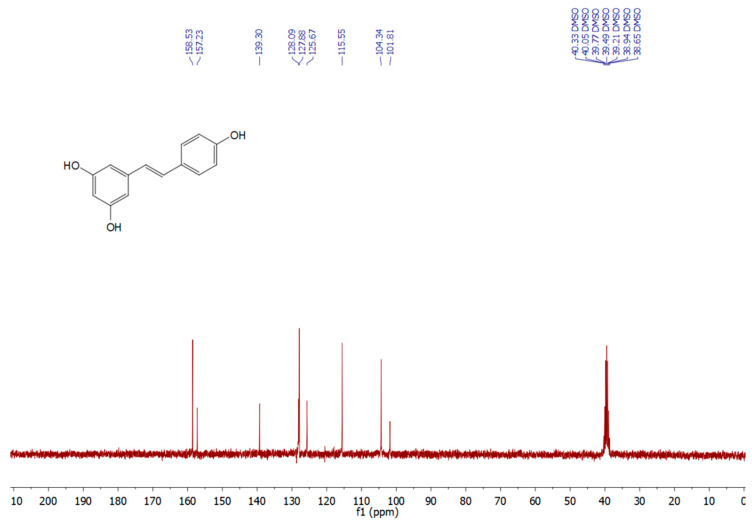
^13^C-NMR of resveratrol (DMSO-*d*_6_, 75MHz).

The endophyte *A. alternata* was cultivated on potato dextrose broth (PDB) medium for 7 days. Fermentation broth was extracted with ethyl acetate (EtOAc) and RES was isolated as a main secondary metabolite. PDB is a common medium for fungal cultivation. Several grapevine-derived fungal endophytes exhibited the ability to produce or biosynthesize resveratrol when growing on PDB medium, *Aspergillus stellifer* AB4, *Alternaria* sp. AB5, and *Penicillium* sp. AB10 [4], *Xylaria psidii* [35], *Botryosphaeria* sp. [3], indicating the established role of PDB as a baseline medium for RES production and other bioactive secondary metabolites. RES was obtained as a grayish to off-white powder. The amount of RES content was estimated by HPLC using standard RES chromatogram (Figures S1 and S2). We found that our isolate *A. alternata* produced 8.25 mg/L. RES production by endophytes largely depends on the strain type, culture conditions, and presence or absence of elicitors [5]. *Alternaria* sp. MG1 produced 104 μg/L RES after cultivation on GA1 medium for 7 days [31], this amount was significantly improved up to 240.57 μg/L upon UV radiation [36]. Here, the amount of RES produced is significantly higher compared with previously reported data, *A. stellifer* AB4 288 μg/L [4], *Botryosphaeria* sp. 288 μg/L [31], *A. alternata* SK. 206 μg/L [34]. On other hand, from 240 mg/L of crude EtOAc residue/liter of medium, an amount of RES (40 µg) was recovered from *Vitis vinifera*-derived fungus *Quambalaria cyanescens* [37]. Our findings identified the present strain as a candidate for commercial RES production. The structure of RES was elucidated and identified depending on NMR techniques. ^1^H and ^13^C NMR spectral analyses are shown in Figure 2 and Figure 3. The ^1^H and ^13^C NMR spectra data (Table 1) confirmed the chemical structure of RES and revealed signals characteristic of a stilbene backbone with two aromatic rings and phenolic substitutions, consistent with previously reported data [38,39].

### 2.3. Characterizations of RES-AgNPs

#### 2.3.1. Visual Observation on RES-AgNPs Biosynthesis

**Figure 4:**
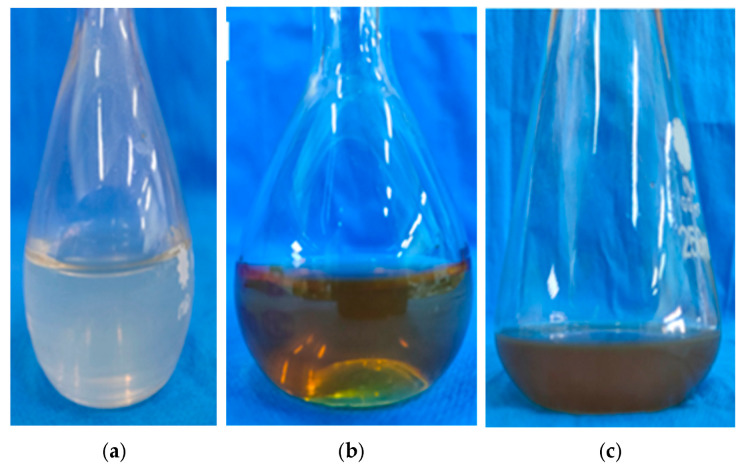
Visual observation of (**a**) AgNO_3_ solution, (**b**) RES extract solution, and (**c**) RES-AgNPs (after 2 h).

The observation of color change is a preliminary test that revealed the efficacy of the RES extract in AgNP fabrication (Figure 4). The development of a dark brown color in the reaction mixture confirmed the reduction of silver ion to metallic silver and successful synthesis of RES-AgNPs [41]. Initially, the reaction mixture showed no color change and turned to a dark brown color after 2 h of incubation, revealing the AgNP formation. The precipitation of AgNPs at the bottom of the conical flask confirmed the completion of silver metal reduction. Green synthesis of AgNPs is a sustainable strategy that minimizes cytotoxic effect and enhances biological activity compared to conventional routes [42]. RES can be employed for the green synthesis of AgNPs, performing a dual role: reducing Ag^+^ to Ag^0^ and acting as a capping agent, resulting in the formation of a stable and biocompatible structure of RES-AgNPs [43]. This combination maximizes the benefits of both partners and overcomes the obstacles of individual application. Conjugating RES-AuNPs and RES–AgNPs showed significant antibacterial potential against both Gram-negative and Gram-positive bacterial strains, compared with the application of RES alone [44]. Furthermore, RES-AgNPs exhibited greater effects in alleviating oxidative stress and inflammatory response in a rat model, compared with RES alone [29].

#### 2.3.2. UV–Vis Spectroscopy

**Figure 5:**
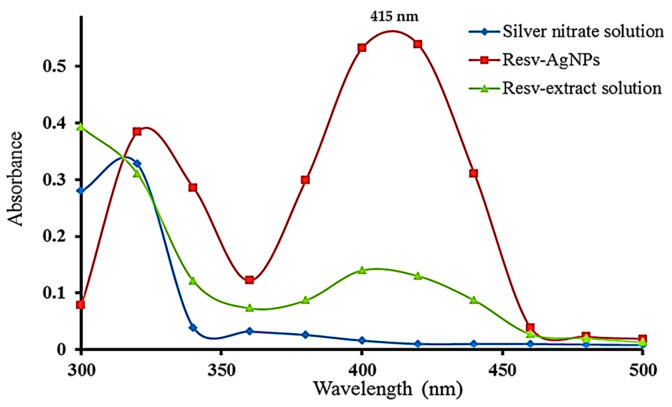
UV–visible spectra of AgNO_3_ solution, RES-extract ethanolic solution, and the synthesized RES-AgNPs after 2 h (*n* = 3).

The UV–Vis spectroscopy analysis is a simple and reliable technique used to monitor and ensure the presence of synthesized AgNPs. UV–Visible spectra were obtained for the AgNO_3_ solution, RES extract solution, and the synthesized RES-AgNPs. Deionized water was used as a control for each investigated sample. Peaks in the region of 380–450 nm for the samples are shown in Figure 5. According to the previous documented results, RES-AgNPs are showing a plasmon resonance absorption band due to the surface plasmon vibration excitation at 415 nm in the UV–visible spectrophotometric analysis [45]. The sharpness of the NPs peak indicated the synthesis of small-sized particles. Consistent with our finding, Park et al. [44] recorded surface plasmon vibration for prepared RES-AgNPs at 412–417 nm.

#### 2.3.3. Particle Size, Polydispersity Index, and Zeta Potential

**Figure 6:**
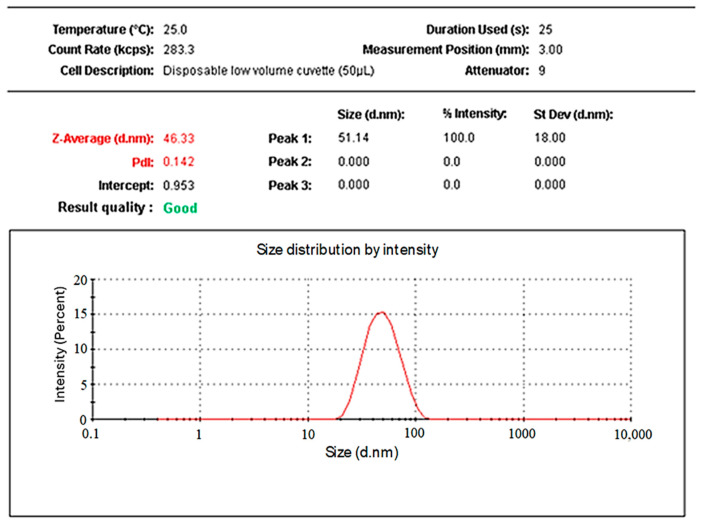
Particle size distribution of the synthesized RES-AgNPs.

Dynamic light scattering (DLS) was utilized to determine the particle size, polydispersity index (PDI), and zeta potential of the synthesized AgNPs in a colloidal aqueous media. As depicted in Figure 6, the particle size distribution of RES-AgNPs was 46.07 ± 4.16 nm (*n* = 3). The particles size of AgNPs fluctuated and ranged from 1 to 100 nm [42]. A reduced particle size of AgNPs increases the surface area-to-volume ratio, thereby enhancing their chemical reactivity and improving their potential interactions with biological systems [46]. RES-AgNPs prepared with NaOH exhibited smaller particle size 11.5 ± 3.18 nm, suggesting the reduction role of NaOH on particles [44]. Conjugating RES with previosly prepared AgNPs expanded the particle to a range of 95 to 160 nm [29]. The PDI value of synthesized RES-AgNPs was 0.144 ± 0.064 (*n* = 3). The PDI value represents monodisperse particle distribution whereas a value less than 1 represents homogenous particles distribution [47]. Narrow size distribution (PDI value < 0.5) of RES-loaded nanocarrier has been recorded in several reports [48,49].

**Figure 7:**
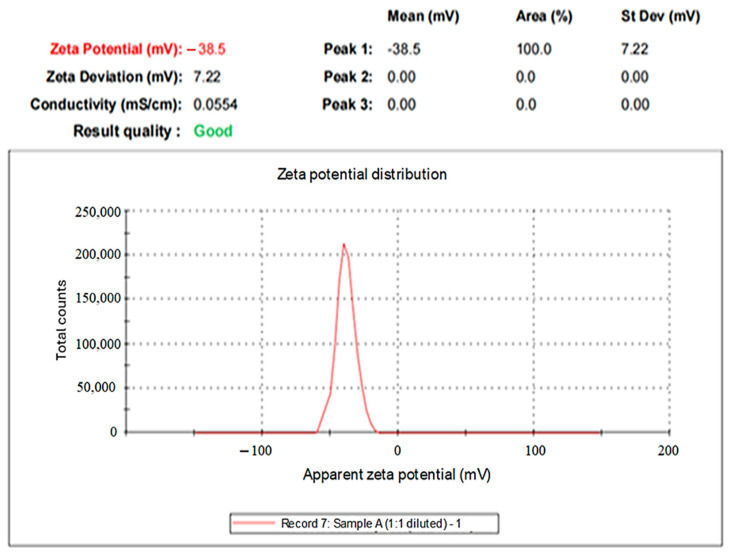
Zeta potential of the synthesized RES-AgNPs.

In the present investigation, the negative zeta potential was found at −38.1 ± 1.24 mV (*n* = 3) (Figure 7). High absolute negative zeta potential value indicates on the surface of the AgNPs, has a high electrical charge which could generate a strong repulsive force between the particles to prevent their aggregation and support their stability and biological interactions [50]. It has been reported that RES-conjugated with AgNPs slightly increase the negative charge of particles surface from −42.6 mV to −45.4 mV [29]. Our findings are in line with the study reported by Park et al. [44], where they measured zeta potentials at −37.49 ± 1.65 mV for RES-AgNPs prepared with NaOH. This result confirmed successful surface conjugation of RES with AgNPs forming electrostatic stable structure.

#### 2.3.4. X-Ray Diffraction (XRD)

**Figure 8:**
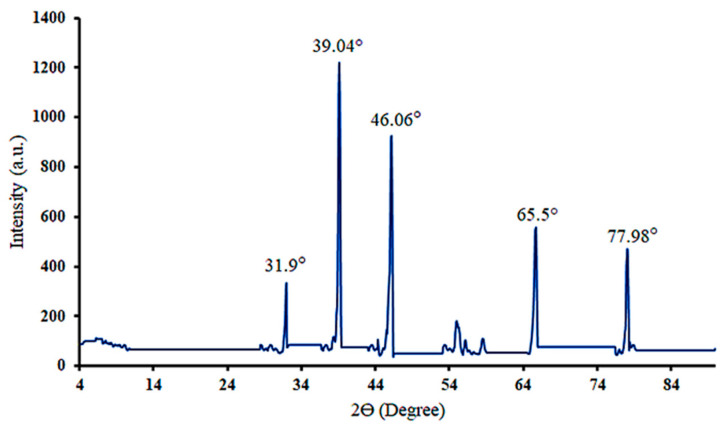
XRD pattern of synthesized RES-AgNPs.

The purity and crystalline structure of the synthesized AgNPs of RES were studied by performing XRD analysis. As illustrated in Figure 8, the XRD pattern of the synthesized RES-AgNPs shows four characteristic peaks at 39.04°, 46.06°, 65.5°, and 77.98°, which are attributed to the (111), (200), (220) and (311) crystallographic planes, respectively, characteristic of face-centered cubic AgNPs [51]. The AgNPs’ crystalline nature and purity of the NPs depended on the position, height, and width of the peaks. In the XRD spectrum of the biosynthesized AgNPs, the sharp peaks correspond to AgNP formation with high crystallinity and small particle size [52]. According to the obtained XRD spectrum, the main peaks that were recorded belong to the AgNPs. There are three more peaks in the diffractogram at 31.78°, 54.88°, and 58.3°. These peaks have been obtained due to AgNO_3_, which might not have been reduced and hence remained in the sample, investigated in a small quantity [53].

#### 2.3.5. Particle Size Calculation Using the Scherrer Equation

For the most intense diffraction peak located at 2θ = 39.04°, the Bragg angle, θ = 19.52°. The measured FWHM (β) = 0.17052° (using OriginPro program, version 10.3.0.197).

By conversion of β to radians, β = 0.17052 × π/180 = 0.00298 radians.

Then, substitute the obtained values in the Scherrer equation:

$$D\;=\;\frac{0.94\times 0.15418}{0.00298\times \cos\left(19.52\right)}=\;\frac{0.13865}{0.00280}=51.6312\;\mathrm{nm}.$$

Thus, the average crystallite size of the prepared RES-AgNPs is 51.6 nm.

#### 2.3.6. FT-IR Spectroscopy

**Figure 9:**
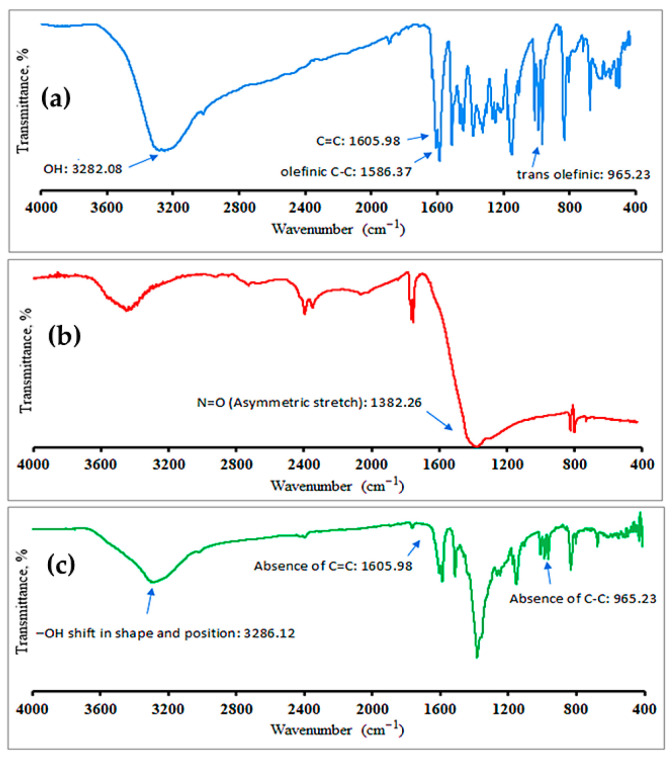
FT-IR spectra of (**a**) RES extract, (**b**) AgNO_3_, and (**c**) synthesized RES-AgNPs.

FT-IR is a technique routinely employed to determine the functional groups and chemical bonds during synthesis process [44]. The FT-IR spectrum of pure RES (Figure 9a) showed characteristic peaks belonging to OH functional groups, stretching of aromatic C=C, olefinic C-C stretching, and typical trans olefinic peak at 3282.08, 1605.98, 1586.37, and 965.23 cm^−1^, respectively [54]. Figure 9b indicated the FT-IR spectra of AgNO_3_. The obtained spectra showed peaks at 3450.52, 2349.60, and 1752.79 cm^−1^ with a band at 1382.26 cm^−1^ due to stretching vibration of N=O [55]. The FT-IR spectrum of the synthesized RES-AgNPs showed noticeable peak shifts, along with changes in intensity and the disappearance of certain peaks, as shown in Figure 9c. The shifted bands revealed that the corresponding functional groups were involved in the reduction in silver ions and the stabilization of the resulting nanoparticles. The observed shifts in the shape and position of the –OH stretching band of RES at 3286.12 cm^−1^ indicate its contribution in the reduction and the stabilization of the resulting nanoparticles. The absence of peaks 1605.98 and 965.23 cm^−1^ clearly indicates that the aromatic C=C and olefinic C-C groups are involved in the stabilization of AgNPs [56].

#### 2.3.7. TEM Analysis

**Figure 10:**
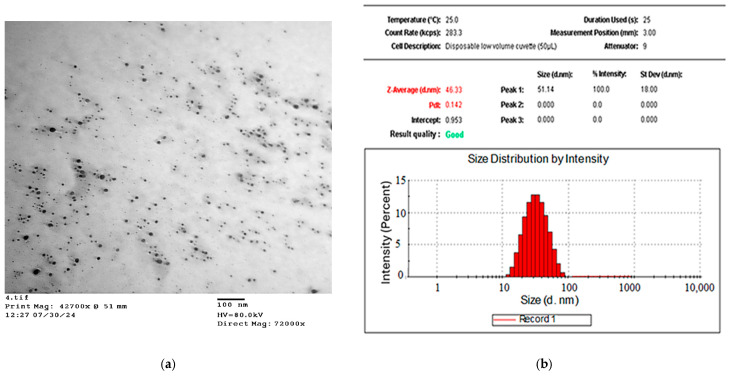
(**a**) TEM micrograph of the synthesized RES-AgNPs, and (**b**) particle size distribution histogram.

TEM analysis was carried out on the synthesized RES-AgNPs to observe the individual size and shape of the AgNPs. A TEM micrograph of the investigated sample is shown in Figure 10a, which is consistent with the UV-Vis absorption spectra results. A large number of NPs were uniformly distributed within a size range of 25 to 47 nm as revealed by the particle size distribution histogram (Figure 10b). This indicates that the distribution of AgNPs stabilized by the RES extract is rather uniform. The synthesized RES-AgNPs are spherical in shape without formation of aggregates due to electrostatic force of repulsion which ensure the stability of the synthesized RES-AgNPs [57]. Our findings were comparable with the previous data. Shukla et al. reported that RES-AgNPs were spherical with particle size ranging from 11 to 21 nm based on the ratio of gold to RES [58]. Meanwhile, the mean size of RES-AuNPs was 14.60 ± 2.97 nm, as observed by high-resolution TEM [44].

### 2.4. RES and RES-AgNPs Selectively Inhibit the Growth of Epidermoid Carcinoma Cells

**Figure 11:**
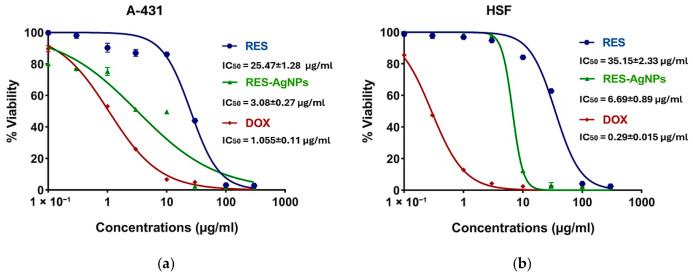
Cytotoxic activity of RES, RES-AgNPs, and doxorubicin (DOX) on (**a**) A-431 cancer cells and (**b**) HSF normal cells after 48 h treatment. Data represent mean ± SD (*n* = 3).

**Figure 12:**
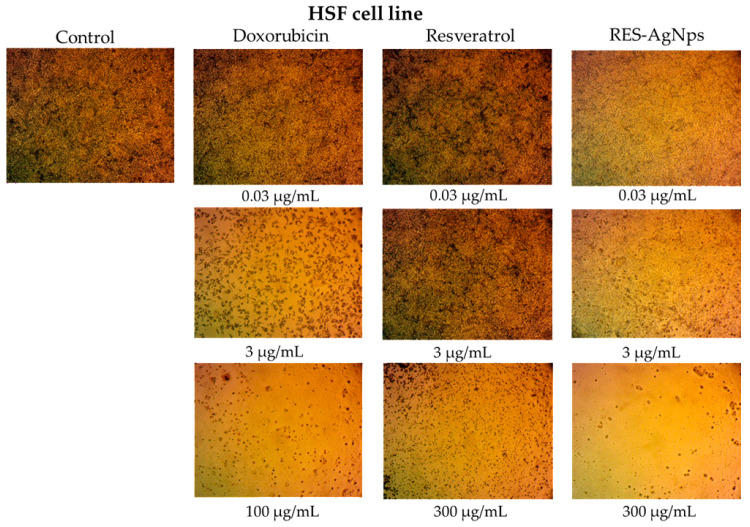
Optical microscope images of cytotoxicity assay of doxorubicin, RES, and RES-AgNPs against HSF cell line. Magnification power: ×100.

**Figure 13:**
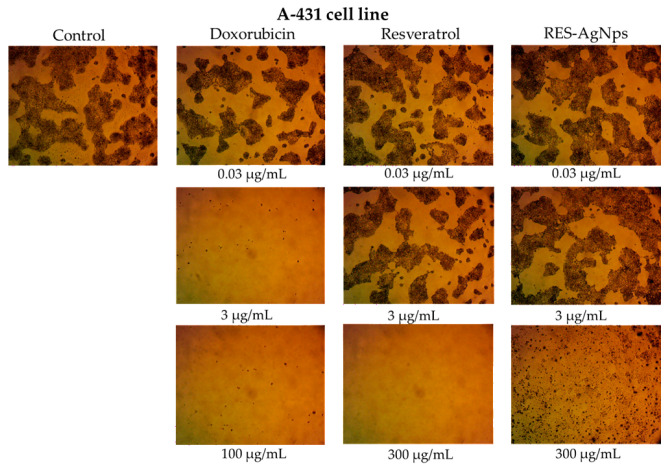
Optical microscope images of cytotoxicity assay of doxorubicin, RES, and RES-AgNPs against A-431 cell line. Magnification power: ×100.

Skin cancer comprises a spectrum of malignant neoplasms, with incidence and mortality rates increasing continuously, contributing to a substantial increase in its global burden [59,60]. Accordingly, this study aimed to evaluate the cytotoxic activity and cancer cell specificity of RES and its complex with AgNPs. Both epidermoid cancer cells (A-431) and normal human skin fibroblast cells (HSF) were exposed to RES and RES-AgNPs for 48 h, followed by cell viability assessment using the MTT assay. Both RES and RES-AgNPs exhibited concentration-dependent cytotoxicity, and reductions in viability observed at concentrations of 10 and 30 μg/mL, respectively (Figure 11a,b). In A-431 skin cancer cells, the calculated IC_50_ values were 25.47 ± 1.28 μg/mL for RES and 3.08 ± 0.27 μg/mL for RES-AgNPs. Interestingly, in HSF normal cells, IC_50_ values were 35.15 ± 2.33 μg/mL for RES and 6.69 ± 0.89 μg/mL for RES-AgNPs. These results correspond to selectivity index (SI) values of 1.4 for RES and 2.17 for RES-AgNPs, indicating that RES-AgNPs are more selective for cancer cells (Figure 12). Doxorubicin (DOX), used as positive control in this study, exhibited pronounced cytotoxic activity against A-431 cells with an IC_50_ of 1.055 ± 0.11 μg/mL (Figure 11b). However, it showed a more potent cytotoxicity in HSF normal cells, with an IC_50_ of 0.29 ± 0.015 μg/mL, indicating lower cancer cell specificity (SI) < 1 (0.27) (Figure 13). Selectivity index (SI) values greater than 1.00 indicate that the tested substance exhibits higher toxicity to cancer cells than normal cells, reflecting increased selectivity [61] and implying that a lower concentration of the substance is required to kill cancer cells than normal cells.

The cytotoxic properties of RES toward A-431 cancer cells are well documented in several studies. RES can promote A-431 cells apoptosis and cell cycle disruption through suppression of NF-κB signaling pathway and modulation of JAK/STAT [12,62,63,64]. In line with previously reported studies, the therapeutic efficacy of RES was improved when combined with nanocarriers [65,66].

RES-AgNPs showed enhanced cytotoxic activity, greater selectivity, and low toxicity toward normal cells, highlighting the potential of NPs to improve the pharmacological effects of natural compounds such as RES [67]. Our findings highlight one of the main drawbacks of conventional chemotherapeutics, their lack of selectivity and associated toxicity toward normal cells, despite their potent cytotoxic activity [68,69].

In the absence of previous standardized formulations and assay conditions across NP investigations, direct numerical comparison of efficacy, stability, and toxicity properties remains limited. However, when positioned within the performance ranges commonly reported for lipid-based, polymeric, and hybrid NPs, our RES-AgNPs formulation exhibits characteristics consistent with well-optimized delivery systems. For instance, the obtained enhanced cytotoxic responses are broadly in line with reports showing that NP encapsulation improves cell uptake and therapeutic potency in advanced lipid–polymer hybrid and lipid NP formulations, which are engineered for improved drug bioavailability and controlled release [70]. Similarly, the colloidal stability of the fabricated RES-AgNPs was evidenced by narrow size distribution and resistance to aggregation over the storage period, corresponds with stability behaviors described for lipid-based and hybrid NPs designed to maintain structural integrity during storage and biological exposure [71]. While methodological heterogeneity across previous investigations prevents direct quantitative equivalence, these qualitative comparisons collectively place the present RES-AgNP formulations within the expected performance profile of modern NP analogs [72].

**Figure 14:**
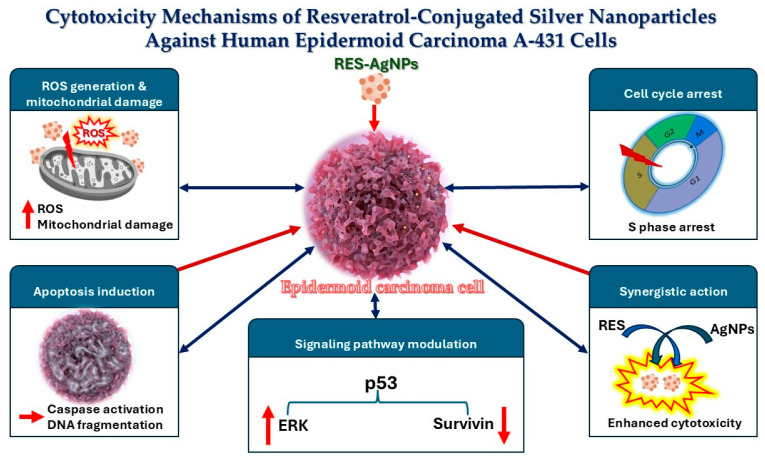
Cytotoxicity mechanisms of RES-conjugated AgNPs against A-431 epidermoid carcinoma cells.

Resveratrol-loaded silver nanoparticles possess potent cytotoxic effects on A-431 skin cancer cells via several pathways (Figure 14) including: (i) Reactive oxygen species (ROS) generation, which induces mitochondrial damage, disrupts membrane potential, and activates apoptotic pathways. (ii) Triggering cell cycle arrest, primarily in the S phase. (iii) Induction of apoptosis by suppressing telomerase activity and downregulating human telomerase reverse transcriptase (hTERT) expression, both of which are essential for sustained tumor cell growth. (iv) The synergistic interaction enables controlled and sustained drug release, boosting bioavailability and shielding the compound from rapid degradation, resulting in higher overall cytotoxic efficacy compared to free resveratrol. (v) Modulation of key signaling pathways, such as upregulating p53 and extracellular signal-regulated kinase (ERK) while downregulating survivin, collectively contributes to effective tumor suppression [67,73].

To sum up, the possibility for continuous, climate-independent production is what makes this source economically significant, even though the current laboratory-scale yield of *A. alternata* reached 8.25 mg/L. Industrial fermentation costs scale nonlinearly, in contrast to the 1000 USD/200 mg price of analytical-grade commercial standards. It is anticipated that future nitrogen source improvement and the use of elicitors (such methyl jasmonate) would close the gap between biological novelty and economic feasibility.

## 3. Materials and Methods

### 3.1. Plant Material and Fungal Isolation

Endophytic fungus *A. alternata* was isolated and identified from the stem of grapevine *Vitis vinifera* L. cultivar prime. Plant (*V. vinifera*) was identified by plant taxonomy staff members based on reference books with species descriptions, keys, and illustrations. A plant voucher specimen was deposited at the herbarium of the Botany and Microbiology Department, Faculty of Science, Al-Azhar University, Assiut, Egypt, with a voucher number (ABH695-74.19). Isolation of endophytes was carried out according to the established procedure by Dwibedi and Saxena [3]. Plant materials were rinsed in sterile distilled water to remove dust on the plant surface and cut into small segments using a sterile scalpel, then immersed in 70% ethanol (AL-Nasr Chemicals Co., Cairo, Egypt) for 30 s followed by 1% sodium hypochlorite (AL-Nasr Chemicals Co., Cairo, Egypt) for 2 min, and then washed 3 times with sterilized distilled water and placed on potato dextrose agar (PDA, HiMedia, Maharashtra, India) medium under aseptic conditions. Petri plates were incubated at 28 °C for 7 days. The emergence hyphae were transferred to new PDA plates and re-cultured several times under the same condition to ensure the purity of fungal isolate. The purified isolate was maintained with 50% glycerol and kept at −20 °C for future investigation. Five endophytic fungi were purified where *A. alternata* was selected in this study upon its highest RES production (Table S1).

### 3.2. Molecular Identification of the Fungus

Molecular identification of the endophytic fungus *A. alternata* was conducted by sequencing the internal transcribed spacer (ITS) region, followed by aligning the obtained sequence using the BLAST+ 2.17.0 search tool with the existing sequences in NCBI database (http://www.ncbi.nlm.nih.gov/BLAST/ (accessed on 13 March 2025)) to determine their similarity. Briefly, total DNA was extracted from 5-day-old fungus culture employing Norgen Plant/Fungi DNA Isolation Kit, Sigma, Thorold, ON, Canada [74]. According to Mohamed et al. [75], the ITS region was amplified using the forward primer ITS-1 (5′-TCC GTA GGT GAA CCT GCG G-3′) and reverse primer ITS-4 (5′-TCC TCC GCT TAT TGA TAT GC-3′), PCR setting was adjusted according to Hassan et al. [76]. Sequences similarity was analyzed by BioEdit software program version No. 7.2.5.

### 3.3. Production and Detection of RES

A sterilized PDB medium (HiMedia, India) was inoculated aseptically with 5 mm mycelial plug of endophytic fungus *A. alternata* and incubated in a rotary shaker at 28 °C for 7 days. After incubation, the culture filtrate was collected using Whatman filter paper. The culture supernatant was extracted with double volume of ethyl acetate (EtOAc, AL-Nasr Chemicals Co., Cairo, Egypt). The organic layer was dehydrated and reconstituted in methanol [35].

Resveratrol, in the EtOAc crude extract, was qualitatively assayed using thin layer chromatography (TLC) and further confirmed by high performance liquid chromatography (HPLC). Briefly, 10 µL of endophytic fungus *A. alternata* crude extract was spotted on TLC plate (20 × 20 cm) with 5 μg/mL standard RES solution (Sigma-Aldrich, Schnelldorf, Germany). All spots were developed on the plate with the function of mobile system ethyl acetate: methanol: water (Analytical Grade, Alpha Chemika, Mumbai, India) in a ratio of 8:1:1, *v*/*v* respectively. The separated bands were visualized under a UV light chamber; the characteristic blue fluorescence spot of RES under UV was compared with that developed from *Alternaria* crude extract. Rate of flow (R*_f_*) value of the two comparable spots was calculated and compared from the following equation: R*_f_* = distance moved from the blue spot center to the baseline/distance moved by the mobile system to the baseline [77].

The EtOAc crude extract was subjected to RES quantitative measurement using Agilent 1260 HPLC (Agilent Technologies, Waldbronn, Germany) equipped with EC-C18 column (150 × 4.6 mm) and diode array detector. The mobile system composed of 50% acetonitrile as eluent A and 0.1% formic acid dissolved in water as eluent B (HPLC Grade, Alpha Chemika, Mumbai, India). The flow rate was 0.4 mL/min with the sample volume of 5 µL. A multistep gradient was adjusted, 0–10 min, 90% eluent A; 10–35 min, 5% eluent A; 35–45 min, 90% eluent A. Operation temperature was set at 30 °C and detected wavelength at 320 nm.

### 3.4. Extraction and Purification of RES

Extraction and separation of RES were carried out according to Liu et al. [77] with some modifications. Following large-scale production, the PDB medium was extracted with ethyl acetate, and the extract was dehydrated using anhydrous sodium sulphate and then concentrated under vacuum at 50 °C to obtain crude extract (27.5 gm). The crude extract was subjected to normal phase silica gel (12 g × 30 μm) flash chromatography (puriFlash XS 520 Plus, Interchim, Montluçon Cedex, France) with gradient elution of mobile phase composed of dichloromethane/methanol gradients (100:0, 95:5, 90:1, 85:15, 80:20, and 0:100 *v*/*v*). The flow rate was set to 15 mL/min at room temperature.

Similar fractions were gathered. The fractions eluted with dichloromethane/methanol (85: 15 *v*/*v*), 8 g, were chromatographed by normal phase silica gel column (12 g × 30 μm) with mobile phase gradient of *n*-hexane/EtOAc (70:30, 65:35, 60:40 and 50:50 *v*/*v*). The fractions eluted with *n*-hexane/EtOAc (50:50, *v*/*v*) (3.25 g) were subjected to silica gel column chromatography (2 × 100 cm, i.d.) eluted with *n*-hexane/EtOAc (50:50, *v*/*v*) (Analytical Grade, Alpha Chemika, Mumbai, India), isocratically, to afford RES compound (206.25 mg). Chromatographic isolation was performed with the aid of flash chromatography and column chromatography employing silica gel 60 (Merck, Darmstadt, Germany); meanwhile, TLC analysis was established using silica gel precoated plates F_254_ (Merck, Darmstadt, Germany). After that, solvent was evaporated and the purified RES was quantified.

### 3.5. NMR Analysis

^1^H- and ^13^C-NMR analyses for purified RES were recorded in dimethyl sulfoxide (DMSO-*d*_6_) as deuterated solvent using Varian mercury 300 MHz (Varian, Inc., Palo Alto, CA, USA) and were referenced to TMS (*δ* scale) with 300 and 75 MHz as operating frequencies, respectively.

### 3.6. Preparation of RES-AgNPs

Accurately weighed purified RES (200 mg) was dissolved in ethanol (20 mL) (Loba Chemie, Mumbai, India) utilizing ultrasonic water bath (K-SONIC, Gyeonggi-do, Republic of Korea) for 15 min. After that the extract solution was filtered by hydrophobic membrane filter with 0.2 µm pore diameter (Versapor, German Sciences, Freiburg, Germany) (Solution A). In a separated glass beaker, silver nitrate (AgNO_3_) solution (Alpha Chemika, Mumbai, India) was prepared by dissolving 0.0339 gm in 20 mL deionized water (0.01 M) (Solution B). In conical flask, solution B was charged and solution A was added (1 mL/min) utilizing a syringe in ratio of 5:1 *v*/*v* using syringe system and the reaction mixture was heated using thermo-controlled magnetic stirrer MSH-300 at speed of 500 rpm (Biosan, Riga, Latvia) in closed vessel to avoid oxygen and carbo dioxide effects at 60–70 °C for 2 h. The pH was adjusted to be 7 ± 1 using 0.1 M acetic acid (AL-Nasr Chemicals Co., Cairo, Egypt). A visible color change from pale yellow to dark brown was observed, which is indicative of the successful formation of AgNPs.

The RES-AgNPs preparation mechanism could be controlled by the reduction in Ag^+^ ions by the polyphenolic structure of RES, which acts simultaneously as a reducing and stabilizing agent [78]. Upon mixing RES solution with the AgNO_3_ solution, the -OH groups of RES donate electrons to Ag^+^, generating Ag^0^ atoms and initiating prompt nucleation [79]. As nucleation calms down, the remaining Ag ions are progressively reduced and deposited onto the first formed nuclei, driving controlled NP growth. The utilized ratio of RES to Ag^+^ plays a focal role in directing these processes. The preliminary tests showed that the higher RES ratios enhance the reduction rate and promote the formation of a larger number of nuclei, yielding smaller and more uniform NPs, whereas lower RES ratios slow the nucleation rate and support extensive growth, resulting in larger NPs [80]. In addition, the aromatic rings and -OH groups of RES could be adsorbed onto the NP surface, providing capping and steric stabilization that help define morphology and prevent agglomeration of the obtained AgNPs. These mechanistic considerations account for the observed particle size distribution and structural characteristics obtained under our optimized synthesis conditions.

The obtained mixture was further stirred for 24 h at room temperature in dark conditions before lyophilization (Freeze dryer, Acculab, FD55-10S, New York, NY, USA) [81]. The obtained lyophilized RES-AgNPs were stored in desiccator until further characterization.

### 3.7. Characterizations of RES-AgNPs

The synthesized RES-AgNPs were characterized by different methods as described by Abdallah et al. [82].

#### 3.7.1. Ultraviolet–Visible Spectroscopy (UV-Vis)

The reduction in Ag^+^ ions was monitored by withdrawing aliquots (4 mL) of the reaction mixture at 24 h intervals and recording their UV–Vis spectra using a double-beam UV–Vis spectrophotometer (UV-1601, Shimadzu Co., Kyoto, Japan). To determine the maximum absorbance (λ_max_), spectral scanning was performed by measuring the optical density over a wavelength range of 200–800 nm [83].

#### 3.7.2. Particle Size, Polydispersity Index, and Zeta Potential

The average mean of particle size (PS), PDI, and zeta potential (ZP) of RES-AgNPs were determined using DLS with a Malvern Zetasizer (ZEN 1690, Nano-S90; Malvern Instruments Ltd., Worcestershire, UK). Measurements were carried out at room temperature (25 °C) at a scattering angle of 90°, after suitable dilution of the samples [84]. The measuring procedure was conducted in triplicate manner, and the data was expressed as the mean ± the standard deviation (SD).

#### 3.7.3. X-Ray Diffraction (XRD)

The XRD pattern of RES-AgNPs was obtained using a powder diffractometer Philips 1710 diffractometer (Philips, Almelo, The Netherlands) with Kα radiation. The equipment was operated at a voltage of 40 kV and a current of 30 mA in the 2ϴ range from 20 to 90°. The purity, size, and crystalline behavior of the prepared RES-AgNPs were analyzed by recording the XRD pattern [85]. The average crystallite size was calculated using the Scherrer equation based on the most intense diffraction peak [86].

D (nm) = Kλ/β × cos θ

where D is the crystallite size in nm, K is the dimensionless shape factor (taken as 0.94), λ is the X-ray wavelength (=0.15418 nm), β is the full width at half maximum (FWHM) of the selected diffraction peak (in radians), and θ is the Bragg angle corresponding to the peak position (peak center).

#### 3.7.4. Fourier-Transform Infrared (FT-IR) Spectroscopy Analysis

FT-IR spectroscopy analysis was employed to determine the possible functional groups responsible for the reduction of silver ions to AgNPs. The sample (RES extract, AgNO_3_, and RES-AgNPs) was analyzed using FTIR spectrophotometer (IR-476-Shimadzu, Kyoto, Japan). Spectra were collected from 6 scans at a resolution of 4 cm^−1^ in the transmission mode of 4000–400 cm^−1^ [87].

#### 3.7.5. Transmission Electron Microscopy (TEM) Imaging

RES-AgNPs morphology was examined using transmission electron microscopy (TEM) (JEM 100 CX, Osaka, Japan) operated at an accelerating voltage of 80 kV. Prior to imaging, the sample was diluted with distilled water, sonicated for 5 min to ensure proper dispersion, and then allowed to dry at room temperature [88].

### 3.8. Cytotoxicity Assay

#### 3.8.1. Cell Lines and Culture Conditions

Human A-431 carcinoma cell line, and HSF normal cells were obtained from Nawah Scientific (Mokattam, Cairo, Egypt) and cultured in DMEM (Dulbecco’s modified eagle’s medium, Gibco, NY, USA), supplemented with fetal bovine serum (FBS, Gibco, USA) at a concentration of 10% and 100 U/mL of penicillin and streptomycin (PS, Corning, Glendale, AZ, USA). The cells were incubated at 37 °C in a humidified environment that contained 5% CO_2_ [89,90].

#### 3.8.2. Assessment of Cytotoxicity by MTT Assay

Cell viability was assessed using MTT assay. Briefly, 100 μL of cell suspension (5 × 10^3^ cells) was seeded in 96-well plates (Greiner Bio-One, Kremsmünster, Austria) and incubated for 24 h at 37 °C in a humidified atmosphere containing 5% CO_2_. The cells were treated with 100 μL of medium containing various concentrations (0.03–300 µg/mL) of RES or RES-AgNPs and (0.03–100 µg/mL) of doxorubicin (Dox). After 48 h of treatment, the culture medium was aspirated and 20 μL of MTT (Merck, Rahway, NJ, USA) solution (1 mg/mL) with 100 μL of phosphate buffer solution (PBS, Elabscience, Wuhan, China) was added to each well, followed by incubation at 37 °C for 4 h. The formazan crystals were solubilized in 100 μL of absolute DMSO (Merck, USA), and the absorbance was measured at 570 nm using a multi-well plate reader [91].

Cell viability was calculated using the following formula:

$$\mathrm{Cell}\mathrm{viability}=\left[\frac{\mathrm{Control}\mathrm{OD}-\mathrm{Sample}\mathrm{OD}}{\mathrm{Control}\mathrm{OD}}\right]\times \;100$$

where OD refers to the optical density the control refers to the negative control, representing untreated cells. The IC_50_ values were calculated for each experiment. IC_50_ values were reported as mean ± SD. The selectivity index (SI) was stated as SI = IC_50_ of normal cells/IC_50_ of tumor cells [92].

### 3.9. Data Analysis

Dose–response curves were generated using GraphPad Prism software version 9.3.1(471). Data are presented as means ± SD (*n* = 3).

## 4. Conclusions

Here we identified the grape-derived endophytic fungus *A. alternata* AUMC 16209 as a reliable source for sustainable RES production, an alternative to plant extraction. Purified RES was utilized as a reducing agent for the synthesis of AgNPs, minimizing chemical input. A RES-conjugated nanocarrier system improves the cytotoxic activity toward human epidermoid carcinoma A-431 cells. Microbial-derived resveratrol represents a renewable and potentially scalable alternative that reduces reliance on plant sources and enables long-term cost reduction upon process optimization. The amount of RES produced in this study by *A. alternata* AUMC 16209 is significantly higher compared with some reported species. However, future optimization of fermentation conditions and downstream processing may improve economic feasibility. Further work is required to optimize the RES production by *A. alternata* AUMC 16209, to elucidate the cytotoxicity mechanisms of RES-AgNPs, and to dictate the function of RES-AgNPs toward biological systems. Moreover, further investigation, including detailed pharmacokinetic, toxicological, and in vivo studies, should be done before considering clinical relevance.

## Figures and Tables

**Table 1:** NMR data of the purified resveratrol.

Assignment	*δ*_H_, Multiplicity (*J* in Hz)	Ref. [40]	*δ*_C_ in ppm	Ref. [40]
1			139.30	139.4
2	6.38 (*d*, *J* = 2.1 Hz)	6.38 (*d*, *J* = 1.6 Hz)	104.34	104.4
3			158.53	158.6
4	6.11 (*t*, *J* = 2.1 Hz)	6.11 (*t*, *J* = 2.0 Hz)	101.81	101.8
5			158.53	158.6
6	6.38 (*d*, *J* = 2.1 Hz)	6.38 (*d*, *J* = 1.6 Hz)	104.34	104.4
1′			128.09	128.1
2′	7.35 (*d*, *J* = 8.5 Hz)	7.34 (*d*, *J* = 8.4 Hz)	127.88	127.9
3′	6.75 (*d*, *J* = 8.5 Hz)	6.75 (*d*, *J* = 8.4 Hz)	115.55	115.6
4′			157.23	157.3
5′	6.75 (*d*, *J* = 8.5 Hz)	6.75 (*d*, *J* = 8.4 Hz)	115.55	115.6
6′	7.35 (*d*, *J* = 8.5 Hz)	7.34 (*d*, *J* = 8.4 Hz)	127.88	127.9
7′	6.94 (*d*, *J* = 16.3 Hz)	6.93 (*d*, *J* = 16.4 Hz)	127.88	127.9
8′	6.81 (*d*, *J* = 16.3 Hz)	6.87 (*d*, *J* = 16.4 Hz)	125.67	125.7
3-, 5-OH	9.16 (2H, s)	9.24 (2H, s)		
4′-OH	9.50 (1H, s)	9.60 (1H, s)		

## Data Availability

The original contributions presented in this study are included in the article/Supplementary Material. Further inquiries can be directed to the corresponding authors.

## Supplementary Materials

The following supporting information can be downloaded at: https://www.mdpi.com/article/10.3390/ph19050656/s1, Figure S1: HPLC chromatogram of resveratrol standard at 0.15 mg/mL; Figure S2: HPLC chromatogram of resveratrol in *A. alternata* EtOAc crude extract. Table S1. Fungi isolated and identified from the stem of grapevine *Vitis vinifera* L. cultivar prime with RES (µg/mL) detected by HPLC.
