# Supplementary material for: Mycoendophytic-Derived Green Resveratrol-Conjugated Silver Nanoparticles Inhibit the Proliferation of Human Epidermoid Carcinoma A-431 Cells
Source: Pharmaceuticals (Basel). 2026 Apr 22;19(5):656. doi: 10.3390/ph19050656 (PMC13209862; doi:10.3390/ph19050656)
Supplement: Supplementary file 1 [file pharmaceuticals-19-00656-s001.zip › pharmaceuticals-4258101-supplementary.pdf]

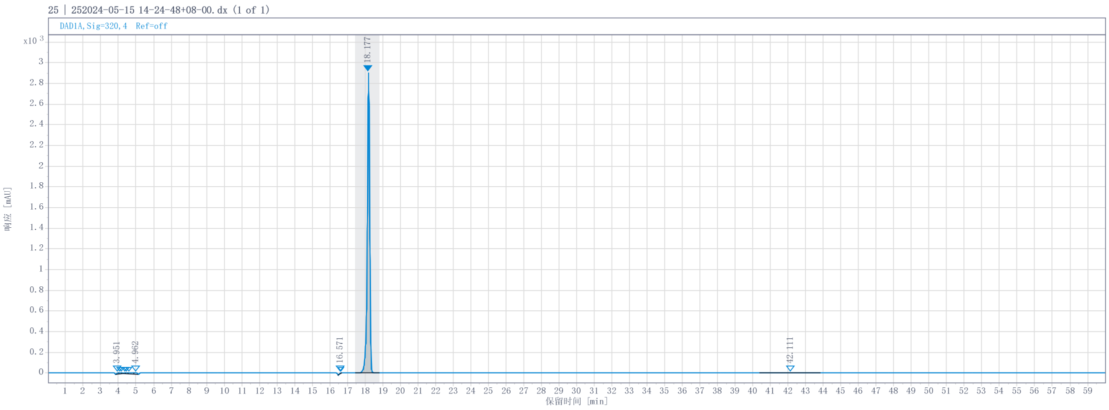

Figure S1. HPLC chromatogram of resveratrol standard at 0.15 mg/mL.

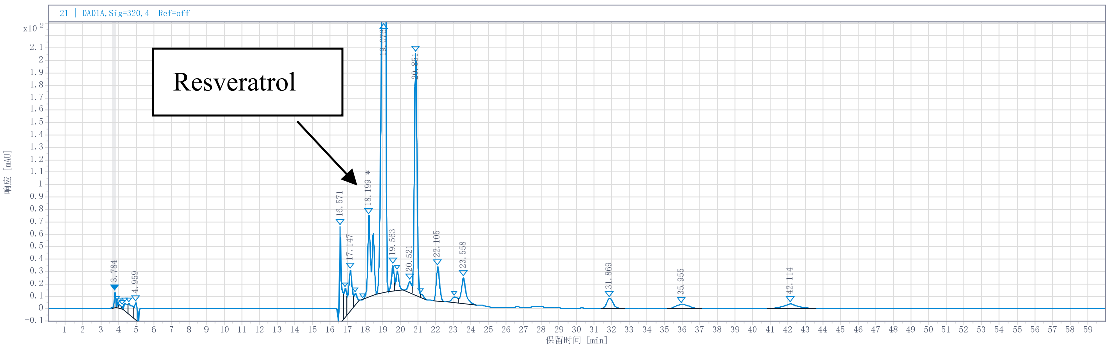

Figure S2. HPLC chromatogram of resveratrol in *A. alternata* EtOAc crude extract.

Table S1. Fungi isolated and identified from the stem of grapevine *Vitis vinifera* L. cultivar prime with RES (μg/mL) detected by HPLC.

**Fungal isolate**	**RES (μg/mL)**
Alternaria alternata	8.25
Aspergillus flavus	ND
Cladosporium sp.	4.50
Penicillium chrysogenum	ND
Trichoderma viride	3.02

ND: not detected
